# Are Lady Almoners Discourteous?

**Published:** 1913-03-01

**Authors:** Edgar S. Kemp

**Affiliations:** Hospital Almoners' Council, Denison House, Vauxhall Bridge Road, S.W.


### Are Lady Almoners Discourteous?

*To the Editor of* The Hospital.

Sir,—I have only a brief comment to make on Mr. Lewcock's further letter. I note that he has substituted the word "discourteous" for the much stronger term which he applied in his first letter to the methods adopted by the lady almoners at certain hospitals. I asked him to mention the number of complaints received, the almoners implicated, and the results of representations to the hospitals concerned. In reply, he quotes three cases. In the first a man was said to have been refused treatment because he earned 30s. a week; apparently this complaint was not referred to the hospital authorities. In the other two instances, which occurred in 1908 and of which no details are given, he "reported them"—I suppose to the hospital or hospitals concerned—but "the results were not encouraging."

I have only to say that no trained almoner adopts, or could fairly adopt, any fixed wage limit and disregard the circumstances of the patient and his application. I must leave your readers to judge whether the evidence Mr. Lewcock has given justifies his contention that the main cause of the dissatisfaction of Hospital Saturday Fund subscribers and of the falling off of their subscriptions is the discourtesy of certain lady almoners.—Yours faithfully, Edgar S. Kemp.

Hospital Almoners' Council,
Denison House, Vauxhall Bridge Road, S.W.,
February 24, 1913.

